# Baseline IL-2 and the AIH score can predict the response to standard therapy in paediatric autoimmune hepatitis

**DOI:** 10.1038/s41598-017-18818-5

**Published:** 2018-01-11

**Authors:** Jana Diestelhorst, Norman Junge, Danny Jonigk, Jerome Schlue, Christine S. Falk, Michael P. Manns, Ulrich Baumann, Elmar Jaeckel, Richard Taubert

**Affiliations:** 1 Department of Gastroenterology, Hepatology and Endocrinology, Hannover Medical School, Hannover, Germany; 2 Pediatric Gastroenterology and Hepatology, Department of Paediatric Kidney, Liver and Metabolic Diseases, Hannover Medical School, Hannover, Germany; 3 Institute for Pathology, Hannover Medical School, Hannover, Germany; 4 Institute of Transplant Immunology, Integrated Research and Treatment Center Transplantation (IFB-Tx), Hannover Medical School, Hannover, Germany; 5 Integrated Research and Treatment Center Transplantation (IFB-Tx), Hannover Medical School, Hannover, Germany

## Abstract

Although autoimmune hepatitis (AIH) can be treated with corticosteroid-based first-line therapy, incomplete remission is associated with progressive liver fibrosis. So far accepted predictors of the subsequent treatment response of AIH patients are lacking. Therefore, we analysed baseline parameters, including iron homeostasis and cytokine levels, in 60 children with paediatric AIH (pAIH). In contrast to adults, elevated serum markers indicating iron overload were not commonly found in children. Therefore, ferritin was not predictive of the treatment response in pAIH. Although baseline immunoglobulins were lower in pAIH children with subsequent complete biochemical remission (BR) upon standard first-line therapy, only lower AIH scores (≤16 points) could predict BR upon standard therapy in our training and validation cohorts. Additionally, higher baseline IL-2 and MCP-1/CCL2 levels were associated with BR in a sub-cohort. A combined score of IL-2 level and a simplified AIH score predicted treatment response more precisely than both parameter alone in this sub-cohort. In conclusion, the baseline AIH score could be validated as a predictor of treatment response in pAIH. Additionally, low baseline IL-2 may help identify children who need salvage therapy. This could be important because the use of low-dose IL-2 therapies is being tested in various autoimmune diseases.

## Introduction

Autoimmune hepatitis (AIH) is a chronic autoimmune liver disease that manifests in all age groups and with an increasing incidence^1^. Paediatric AIH (pAIH) often presents more acutely and has a more aggressive disease course. In addition, the prevalence of AIH type 2 is higher in children and adolescents^2–5^. PAIH has significantly greater overlap with the biliary autoimmune manifestations of autoimmune sclerosing cholangitis (AISC), which are different from those of primary sclerosing cholangitis^3,6^.

Once the diagnosis of active AIH is determined, an immunosuppressive medication that consists of predniso(lo)ne, or alternatively budesonide in non-cirrhotic patients^7,8^, with or without azathioprine, is recommended^9^. Higher predniso(lo)ne doses per body weight are needed to achieve a sufficient treatment response to induction therapy for pAIH compared to adult AIH^9^. Then, biochemical remission rates are similar, approximately 80%, in children and adults^2^.

Since persistent inflammatory activity is associated with histological disease progression and reduced survival in AIH^5,10–12^, the early identification of patients with an insufficient response to standard therapy is clinically important. Recently, we identified dysregulated iron homeostasis and lower immunoglobulin G (IgG) titres as predictors of a good treatment response in adult AIH (aAIH) type 1^13^. Mild iron overload with hyperferritinaemia that was quickly reversible with therapy was associated with complete biochemical remission (BR) upon standard therapy (steroids with or without azathioprine).

Since pAIH differs from aAIH in many clinical aspects, our aim was to identify prognostic baseline markers for the subsequent achievement of BR upon corticosteroid and azathioprine-based first-line therapy in pAIH. Therefore, iron metabolism was systematically assessed at diagnosis and during ongoing therapy and serum cytokines were measured as further immunological markers.

## Results

We retrospectively analysed 60 paediatric patients with untreated, biopsy proven AIH (Table 1, Suppl. Figure 1). Of those patients, 50 reached one of the following treatment endpoints: (1) complete BR under standard therapy (N = 23), (2) incomplete biochemical response under at least two years of standard therapy and/or a switch to salvage therapy due to persistent inflammatory activity (IR; N = 21), and (3) liver transplantation (Ltx; N = 6; after a median of 104 days). The remaining 10 patients either had too short of a treatment duration for classification into BR or IR, or were lost to follow-up. Drug intolerance was not a cause for a switch to salvage therapy in our cohort.

**Table 1:** Baseline data of paediatric AIH patients before initiation of therapy.

	**Overall**		**Responders (BR)**		**Nonresponders (IR + LTx)**		P value
	Median (IQR)	n	Median (IQR)	n	Median (IQR)	n	
Age at diagnosis (years)	13.0 (5.4)	60	12.9 (4.7)	23	12.9 (6.6)	27	0.992
Gender (male/female)	15/45		7/16	23	5/22	27	0.325
AIH type (type 1/type 2)	53/7		21/2	23	24/3	27	1.000
AIH score^a^	19.0 (4.0)	56	16.5 (4.0)	22	19.0 (3.0)	24	0.003
Simplified AIH score^b^	7.0 (1.0)	56	7.0 (1.0)	23	8.0 (1.0)	24	0.021
Follow-up time (months)*			60.3 (85.0)	23	74.2 (98.0)	26	0.133
**Autoantibodies**							
ANA^c^	48	59	16	22	24	27	0.266
SMA^c^	47	59	20	22	20	27	0.159
SLA	6	59	0	22	4	27	0.117
LKM 1^c^	7	59	2	22	3	27	1.000
pANCA	31	59	13	22	13	27	0.445
**Laboratory test**							
IgG (times the ULN)	1.7 (1.1)	59	1.45 (0.80)	22	1.84 (0.87)	27	0.031
Alanine aminotransferase (times the ULN)	10.9 (17.1)	60	15.1 (22.2)	23	9.0 (14.0)	27	0.386
Aspartate aminotransferase (times the ULN)	13.1 (20.2)	60	18.4 (32.7)	23	10.6 (16.0)	27	0.345
Alkaline phosphatase (times the ULN)	1.0 (0.6)	60	1.06 (0.55)	23	1.00 (0.38)	27	0.238
Gamma-glutamyltransferase (times ULN)	3.0 (3.4)	60	3.03 (3.00)	23	2.53 (6.80)	27	0.961
Bilirubin (times the ULN)	1.3 (3.0)	60	1.59 (4.41)	23	1.71 (4.41)	27	0.946
Prothrombin time (%)	72.0 (27.0)	59	72.0 (37.0)	22	73.0 (20.0)	27	0.928
**Iron homeostasis**							
Hb (g/dl)	12.6 (2.4)	50	12.8 (2.7)	22	12.5 (1.8)	22	0.518
Serum iron (µmol/l)	20.5 (19.3)	32	22.0 (22.0)	12	12.5 (20.0)	14	0.252
Transferrin saturation (%)	28.0 (25.0)	31	31.0 (33.0)	11	23.5 (22.0)	14	0.222
Iron binding capacity of transferrin (µmol/l)	69.0 (13.5)	33	71.0 (14.0)	13	66.0 (12.0)	14	0.202
Ferritin (times the ULN)	0.54 (1.17)	34	0.59 (2.2)	14	0.44 (1.0)	14	0.210
**Acute phase reactant**							
CRP (mg/l)	3.0 (4.9)	48	4.0 (4.0)	20	2.0 (5.0)	21	0.624
**Histology**							
mHAI	7.5 (6.0)	50	8.0 (7.0)	19	7.0 (5.0)	22	0.386
Fibrosis (Ishak)	3.0 (3.0)	50	3.0 (2.0)	19	4.0 (3.0)	22	0.458

^a^According to Alvarez *et al*.^17^; ^b^According to Hennes *et al*.^18^, ^c^According to AASLD guidelines 2010^9^. *Documented at our centre according to the AASLD guidelines 2010^9^; BR = biochemical remission; IR = incomplete biochemical remission; Ltx = liver transplantation.

Nine of 21 patients with subsequent IR were converted to a second line therapy. In seven patients the medication used was cyclosporine, one patient with concomitant ulcerative colitis was switched to infliximab and one patient initially received cyclosporine but was then converted to tacrolimus followed eventually by everolimus. Second line therapy was less effective than first line therapy. Only two of nine patients on second line therapy finally reached BR. Twelve children of the 21 children with IR stayed on first line therapy. In two of them azathioprine dosage was adjusted based on 6-methylcercaptopurin and 6-thioguanin levels and clinical side effects. The other ten patients have either had a marginal elevation of aminotransferases (n = 5) or IgG (n = 5). Drug dosages in these ten patients were 1.4 mg/kgBW/d (median, range: 1.1–1.9) azathioprine and 5 mg/d (median, 0–10) prednisolone.

### Iron homeostasis before and with therapy in pAIH

Iron parameters at baseline were associated with the treatment response to standard therapy in adults with AIH^13^. Baseline parameters of iron metabolism were available for 46 untreated paediatric patients (Table 1). Of these patients, 12 (26.1%) were excluded from the evaluation of iron homeostasis due to iron deficiency microcytic anaemia. More iron deficiency anaemia cases were found in the pAIH cohort compared to the adult cohort (2.5%; p < 0.001).

**Figure 1:**
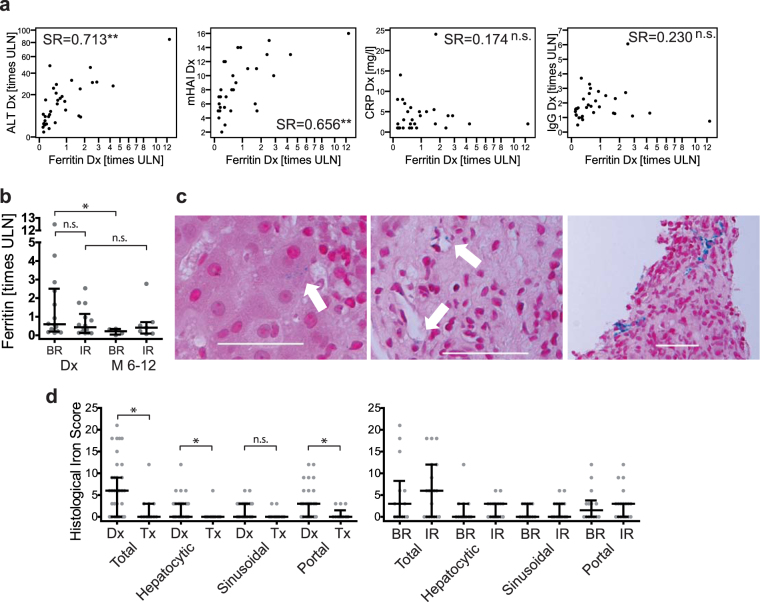
Iron status in untreated paediatric AIH. (**a**) Spearman rank correlation (SR) analysis of serum ferritin in pAIH at diagnosis (Dx) with markers of disease activity, including alanine aminotransferase (ALT, N = 34), C-reactive protein (CRP, N = 31), serum immunoglobulin G (IgG, N = 34) and the modified hepatitis activity index (mHAI, N = 31). (**b**) Serum ferritin in pAIH at diagnosis (Dx) with subsequent biochemical remission (BR) and incomplete response or the need for liver transplantation (IR) and after 6 to 12 months of therapy (M 6-12; treatment duration 9.6 months (median, range: 6.0–11.5)). (**c**) Iron deposition (blue granula) in different compartments of liver biopsies from untreated pAIH patients: (left panel) mostly hepatocellular; (middle panel) the sinusoidal compartment (white arrow); (right panel) portal fields. White horizontal bars represent 50 µm. (**d**) The semiquantitative histopathological iron deposition score in pAIH liver biopsies (left panel) at diagnosis (N = 39) and under therapy (Tx; N = 13) (right panel) at diagnosis, the differences between BR (N = 14) and IR (N = 19) are not significant. (*p < 0.05; **p < 0.01; ***p < 0.001; n.s. = not significant; ULN = upper limit of normal; Horizontal bars represent the median and error bars represent the interquartile range).

In contrast to adults, serum ferritin (SF) and serum markers indicating iron overload were only mildly elevated in children^13^. Abnormal parameters were only observed in a few paediatric patients (hyperferritinaemia in 28% of patients, elevated serum iron (SI) in 25% of patients, and elevated transferrin saturation in 22% of patients; Table 1). Nonetheless, SF was significantly correlated with serum levels of aminotransferases and histological disease severity, but not with CRP or serum IgG (Fig. 1a). During therapy, SF showed a decreasing trend (p = 0.086) and was significantly decreased in patients with BR (Fig. 1b). Similar to adults, we found mild intrahepatic iron deposition in all compartments that regressed with treatment (Fig. 1c,d).

When the treatment response upon standard therapy was considered and children with iron deficiency anaemia were excluded, we found no significant differences in baseline iron parameters between BR and incomplete treatment response patients (IR + Ltx) (Fig. 1b, Table 1). Likewise, intrahepatic iron deposition was not associated with (biochemical) treatment response (Fig. 1d).

SF is an acute phase reactant (APR). However, it was not correlated with other APRs such as CRP (Fig. 1a), IL-1β (SR = 0.000 p = 0.998), IL-6 (SR = 0.058 p = 0.765) or TNF-α (SR = 0.079 p = 0.682). We did not measure hepcidin, the primary regulator of iron homeostasis, because of relevant age-dependent differences, limited studies reporting reference values in children^14,15^, and a sample size that was too small for a sufficient age adjustment in our study. *In-vitro* mouse data showed suppressed hepcidin transcription by hepatocyte growth factor (HGF)^16^. However, there was no correlation of SF with HGF (SR = 0.159, p = 0.409) at baseline in pAIH as found in aAIH^13^.

### The AIH score predicts treatment response in paediatric AIH

**Figure 2:**
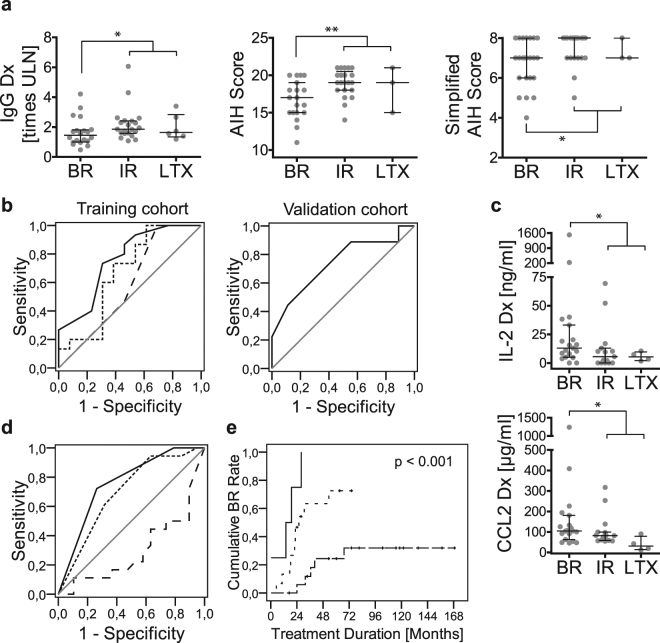
Prediction of treatment response to standard therapy in paediatric AIH. (**a**) Immunoglobulin G (IgG), AIH score^17^ and simplified AIH score^18^ at diagnosis (Dx) in untreated pAIH patients with subsequent biochemical remission (BR), incomplete biochemical response (IR) and the need for liver transplantation (LTX). (**b**) The AUROC analysis for the combined prediction of IR and LTX before the initiation of therapy for IgG (dotted line), AIH score (solid line) and simplified AIH score (dashed line) in the training and validation cohorts. (**c**) Serum levels of IL-2 and MCP-1/CCL2 at diagnosis (Dx) with subsequent BR (N = 19), IR (N = 15) and LTX (N = 4). (**d**) AUROC analysis for the prediction of IR and LTX before the initiation of therapy shown for the simplified AIH score (dotted line), IL-2 (dashed line) and their combined score (black solid line). (**e**) Rates of BR exemplarily shown for the combined score of the simplified AIH score and baseline IL-2 (0 points: black solid line; 1 point: dotted line; 2 score points: dashed line). (*p < 0.05; **p < 0.01; ULN = upper limit of normal; Horizontal bars represent the median and error bars represent the interquartile range).

When all available baseline laboratory parameters were compared regarding the subsequent treatment response (BR vs IR + Ltx), only IgG and AIH scores^17,18^ were significantly higher with IR + Ltx compared to BR (Fig. 2a; Table 1).

To assess the predictive capacity of these differences, the cohort was split into a training cohort (diagnosis until 2010) and a validation cohort (diagnosis since 2010) (Suppl. Table 1). In the training cohort, we performed an AUROC analysis, identified cut-off values guided by Youden’s index and performed a binary logistic regression analysis with these cut-off values. The AIH score was significantly associated with treatment response in the AUROC and binary logistic regression analyses, but IgG and the simplified AIH score were only significantly associated with treatment response in the binary logistic regression analysis (Fig. 2b; Table 2). With cut-off values greater than 1.35 × the ULN for baseline IgG, greater than 16 for the AIH score and greater than 6 for the simplified AIH score, IR + Ltx could be predicted with moderate-high sensitivity but low specificity in the training cohort. The association of a higher AIH score with a worse treatment response (IR + Ltx) was also found with comparable test results in the internal validation cohort (Fig. 2b, Table 3).

**Table 2:** AUROC and univariate analyses for the prediction of treatment response to standard therapy in untreated paediatric AIH.

		AUROC		Binary Logistic Regression			Sensitivity	Specificity
		AUC	Confidence Interval	Cut-off	Odds Ratio	Confidence Interval		
Training cohort	IgG	0.667	0.460–0.873	>1.35 × ULN	8.00	1.32–48.65	0.89	0.50
	simplified AIH score	0.585	0.360–10.808	>6	2.67	1.59–4.47	1.00	0.36
	AIH score	0.762	0.580–10.942	>16	8.00	1.32–48.65	0.89	0.50

(ULN: times upper limit of normal).

**Table 3:** Diagnostic performance of the AIH score to predict incomplete treatment response in untreated paediatric AIH.

Cohort	AIH score	
	Training	Validation
AUC	0.762	0.741
Confidence Interval	0.581–0.942	0.506–0.976
Sensitivity	0.89	0.89
Specificity	0.50	0.44
Positive Predictive Value	0.70	0.62
Negative Predictive Value	0.78	0.80

### Enhanced prediction of the treatment response with baseline IL-2

We also assessed blood cytokine levels at the time of diagnosis (38 samples with subsequent treatment endpoints). The same approach as above was used to predict the treatment response to standard therapy. When we compared the blood levels of 27 cytokines of children with subsequent BR to those with IR + LTx, only baseline IL-2 and MCP-1/CCL2 were significantly different (Fig. 2c). Additionally, we found trends towards higher levels of IL-10, IL-12p70, TNF-α and hepatocyte growth factor (HGF) in the children with subsequent BR (Suppl. Figure 2). Of note, classic B cell cytokines, including IL-4, IL-5, IL-10 and IL-13, did not show significant differences, indicating a stronger impact of the Th1 response.

Due to the small sample number with measured serum cytokines, the cohort could not be split into training and validation sets. Furthermore, the sub-cohort with available cytokine data was too small for a multivariate analysis of IL-2 and MCP-1/CCL2. Therefore, we chose IL-2 for further analyses because of the greater differences between the two treatment response groups (<2-fold difference in the median for MCP-1/CCL2 and > 2-fold difference in the median for IL-2).

Next, the AUROC analysis, Youden’s index and a binary logistic regression analysis for the AIH score, the simplified AIH score, IgG and IL-2 were applied in the sub-cohort with available cytokine data. The simplified AIH score and IL-2 were found to be associated with treatment response to standard therapy in both the AUROC and binary logistic regression analyses (Table 4). When IL-2 was combined with the simplified AIH score for a combined score (sum of IL-2: < 10.8 µg/ml = 1, > 10.8 µg/ml = 0; simplified AIH score: ≤ 6 = 0, > 6 = 1), the predictive capacity in terms of the AUROC and specificity was even higher than with the individual parameters (Table 4, Fig. 2d). Children with a lower treatment response score had significantly higher cumulative treatment response rates (Fig. 2e).

**Table 4:** AUROC and univariate analyses for the prediction of treatment response to standard therapy in a sub-cohort of untreated paediatric AIH with available cytokine measurements.

		AUROC		Binary Logistic Regression			Sensitivity	Specificity
		AUC	Confidence Interval	Cut-off	Odds Ratio	Confidence Interval		
Sub-cohort with cytokine measurements	IL-2	0.701	0.531–0.870	<10.8 µg/ml	5.16	1.23–21.55	0.79	0.58
	IgG	0.654	0.475–0.832	>1.35 × ULN	4.80	1.04–22.10	0.84	0.47
	simplified AIH score	0.696	0.524–0.868	>6	9.92	1.08–91.47	0.94	0.37
	AIH score	0.698	0.527–0.868	>16	3.88	0.84–17.97	0.84	0.42
	Score (simplified AIH score and IL-2)	0.759	0.602–0.916	>1	7.28	1.71–31.08	0.72	0.74

## Discussion

Altered iron homeostasis with elevated serum ferritin, transferrin saturation and serum iron is found in multiple liver diseases beyond haemochromatosis^19^. Hyperferritinaemia is also found in many autoimmune diseases^20^. Furthermore, hyperferritinaemia and lower serum transferrin levels were associated with worse outcome in acute liver failure in a recent study^21^.

In contrast to aAIH, hyperferritinaemia with elevation of other iron parameters, indicating iron overload, was not commonly found in pAIH. Furthermore, baseline hyperferritinaemia was not associated with treatment response in pAIH, but we found higher ferritin and iron levels in aAIH patients with subsequent BR than in those with IR^13^. However, mild intrahepatic iron deposition, detected histologically, was reversible under therapy in both adults and children^13^.

Mechanistically, serum ferritin in pAIH seems more likely to be released from damaged hepatocytes, as implied by the correlation with histological disease severity and the levels of hepatocellular ALT^22^, than to be elevated in the course of an acute phase reaction since there are no correlations with other APRs. In aAIH, we found no stringent evidence for ferritin release from damaged hepatocytes, but we found correlations with some APRs^13^. Although we could further assess iron homeostasis in aAIH via hepcidin measurements, this was complicated in pAIH because of relevant age-dependent differences in hepcidin levels^14,15^ and our cohort was too small to be matched for these variations. Therefore, we did not determine hepcidin levels.

Ferritin is associated with disease severity in terms of aminotransferase levels in pAIH and aAIH^13^. Therefore, higher ferritin levels in aAIH may partially be due to the selection of more severe aAIH cases in our centre (approximately 20-times the ULN of AST and ALT in BR and approximately 15-times the ULN in IR) compared to milder pAIH presentation in this^22^.

We note baseline serum ferritin was normal in about half of the adult patients and in the majority of children with pAIH. Potential explanations that could not be systematically assessed by our studies may be a latent iron deficiency, because one third of children with pAIH had an iron deficient anaemia at initial presentation. Other patients might have had only a subtle increase of ferritin within the normal range. Yet, serum ferritin decreased in 4/5 in paired blood samples despite an initial ferritin level within the normal range.

We chose incomplete biochemical remission to first-line therapy and liver transplantation as a combined endpoint to identify all patients who required closer surveillance or more intense immunosuppressive therapy. As potential predictors for IR, we could identify the following baseline parameters: AIH score, simplified AIH score, IL-2 and MCP-1/CCL2. This is different from the results of Zizzo *et al*. who identified baseline paediatric end-stage liver disease (PELD) and INR as predictors for a switch to second-line therapy in pAIH in a retrospective multicentre study in Canada^23^. The AUC of the INR in the Canadian cohort was comparable to the AIH scores and IL-2 levels in the present study. Compared with the previous literature, the present study differs in the following ways: (I) Our endpoint was any failure to achieve BR as defined by ALT, AST and IgG rather than aminotransferases alone under standard first-line therapy and not the actual switch to second-line therapy. Due to this strict definition, even patients with only marginal and stable elevations of aminotransferases or IgG were considered incomplete responders in the present study. This is the reason why only 9/21 patients with IR were converted to second line therapy. This approach may explain the obvious difference in the treatment response rate between both studies (13% vs 54%). (II) The observation period in the present study was the longest available follow-up (Table 1), but Zizzo *et al*. evaluated the achievement of the endpoint at 24 months after diagnosis. (III) PELD could not be included in the present analysis because the majority of the children were older than 12 years. (IV) In the present study, fewer children (6% vs 15%) had concomitant inflammatory bowel disease. (V) A direct comparison of the INR results would be biased since we could only include prothrombin time because some patients were included before the introduction of the INR. Although we could not confirm the Canadian results, the performance of the AIH scores, which were not included by Zizzo *et al*.^23^, in the Canadian cohort would be interesting to observe. Even though the AIH score and the simplified AIH score are not evaluated for clinical application in pAIH, the scoring system appears to be an appropriate tool for standardised patient characterisation. This is underlined by numerous publications using the AIH scores for pAIH studies and a work of Ebbeson *et al*.^24^.

The AIH score was not associated with treatment response in aAIH at our centre or other centres^13,25^. In contrast, higher baseline IgG was associated with an incomplete response to standard therapy in aAIH patients at our centre^13^, but in pAIH, only a trend towards an association was observed in the AUROC analysis. Other studies have already linked higher IgG levels to worse outcomes or higher relapse rates after the cessation of therapy^26–28^. However, a recently published smaller study that primarily focused on regulatory T cell infiltration in pAIH could not confirm our results and found higher aminotransferase and IgG levels in responders to therapy^29^. However, this study was conducted with a mixed cohort of AIH and autoimmune sclerosing cholangitis patients, two disease groups with remarkable differences. We could at least internally validate our results by objectively splitting our cohort according to the time of diagnosis. Regarding aminotransferase levels, we also observed higher levels in aAIH patients with subsequent BR. Likewise, aminotransferases showed a trend towards higher levels in patients with pAIH who achieved BR. However, the results did not reach significance due to the small sample number and high variability.

The test criteria (AUC, sensitivity and specificity) of the AIH score are moderate and not sufficient to influence a clinical decision alone, but they are within a similar range as the INR in the Canadian cohort^23^ or the IgG and serum ferritin in aAIH^13^. Hopefully, upcoming multicentre initiatives, such as the UK-AIH and the European reference network rare-liver, will provide a basis for more robust predictors than those currently available.

In the further exploration of the predictive potential of peripheral blood cytokines, IL-2 and CCL2 levels were associated with the achievement of BR during standard therapy. CCL2 is a chemokine secreted by monocytes, e.g., in inflamed livers, and liver-infiltrating regulatory T cells (Treg) express CCR4, which binds CCL2. Chemotaxis of CCR4^+^ Tregs by CCL2 could be confirmed *in vitro* ^30^. IL-2, which exhibited greater differences than CCL2 between pAIH responders and non-responders, is an essential survival factor for Tregs and can rescue them from CD95-mediated apoptosis in inflamed livers^31^. Nonetheless, we did not detect significantly higher intrahepatic Treg numbers in patients with subsequent BR in the histological analysis of the present cohort. However, a disproportionate intrahepatic decline in Tregs under therapy was accompanied by a significant decline in IL-2 in the blood^32^. Although blood cytokine levels are not routine clinical parameters, the present results suggested that the predictive capacity of clinical parameters could be improved by non-routine parameters. Furthermore, higher baseline IL-2 levels in children with better subsequent treatment responses are encouraging for the testing of immune-modulating therapy with low-dose IL-2 in AIH. Such approaches have been found to be safe and have shown beneficial effects on Treg homeostasis in a growing number of (auto)immune-mediated diseases such as GvDH, hepatitis C-induced vasculitis, diabetes mellitus type 1 and lupus erythematosus^33–37^.

We found trends towards higher levels of IL-10, IL-12, TNFα and HGF in pAIH children with subsequent BR. The elevation of anti-inflammatory IL-10 may point to more active immune regulatory mechanisms such as IL-2 and CCL2. The elevation of IL-12 and TNFα could imply that more acute AIH manifestations have better treatment responses. The trend towards higher aminotransferases in patients with subsequent BR may support this hypothesis. The trend towards higher HGF levels suggests that other factors, such as tissue regeneration, may also be relevant to the treatment response.

The predictive role of serum IgG levels in adult and paediatric AIH^13,32^ point to a relevant role of the humoral immune response. AIH is considered to be mainly T cell especially Th1 driven^38^ and in our study classical B cell cytokines in the peripheral blood were not predictive for the treatment response. However, the link between the cellular and humoral immune response are follicular T helper cells (Tfh), which were increased in the blood and livers of untreated AIH patients^21,39^. Furthermore, their blood levels were correlated with prognostic serum IgG levels. The correlation of intrahepatic B cell numbers with serum IgG levels in aAIH and the description of intrahepatic lymphoid follicle like structures in autoimmune liver diseases point to an autochthonous production of (auto)antibodies in the liver^40,41^. Since intrahepatic baseline numbers of neither T cells including Treg nor B cells were associated with the subsequent treatment response in adult and paediatric AIH^13,32^, Tfh and their activation state may be crucial for the subsequent treatment response in AIH. However, this association has to be proven directly in further studies.

Taken together, ferritin levels in treatment-naïve pAIH patients do not predict the subsequent treatment response. However, higher AIH scores at diagnosis could be validated internally as a predictor of incomplete treatment response to standard therapy in pAIH. Additionally, lower IL-2 levels may further indicate an incomplete treatment response. This result is important and could promote trials using low-dose IL-2 for AIH, which is currently being tested in many other autoimmune diseases. However, further validation by other centres with larger samples is required before such risk stratification markers can be applied to daily medical care.

## Material and Methods

### Patients

We retrospectively included paediatric patients with untreated, biopsy proven AIH, diagnosed between 1993 and 2015. Children with AISC^3^, replicative viral hepatitis, an AIH score below 10^17^ and bacterial infections at diagnosis were excluded from the study. All laboratory parameters in this study were determined at baseline before the initiation of therapy. The only exceptions were follow-up levels of serum ferritin. These were determined 9.6 months (median, range: 6.0–11.5) after initiation of therapy.

BR was defined as the persistent normalization of aminotransferases (ALT and AST) and immunoglobulin G (IgG) upon first line therapy with steroids (prednisolone, or budesonide as in one child) and azathioprine^9^. First line therapy was based on international guidelines^9,42^. Induction therapy was conducted with 1 mg/kgBW/d (maximum 40 mg/d) prednisolone, which was stepwise reduced under introduction of azathioprine as soon as AST and ALT levels started to decrease. Azathioprine was either started with 1 mg/kgBW/d and be increased after 4 weeks or was directly started with target dose of 1,5 mg/kgBW/d. Azathioprine dose was adjusted depending on metabolite levels, side effects and treatment response. In some cases azathioprine was increased up to 2 mg/kgBW/d (in one single case up to 2,7 mg/kgBW/d). IR was defined as improvements in ALT, AST and IgG without normalization after at least 24 months of first line therapy. IR was not necessarily associated with change to second line therapy. Children with a suspicion of non-adherence were excluded from the evaluation of treatment response. For the development of the predictive treatment response score, our cohort was split into a training cohort (diagnosis until 2010) and a validation cohort (diagnosis since 2010).

This study and all experiments were approved by the local research Ethics Committee of the Hannover Medical School. All experiments were performed in accordance with relevant guidelines and regulations. Written informed consent was obtained from the parents of each child.

### Histology

In addition to the routine pathological review of the liver biopsies, the iron content of the liver biopsies was assessed with the semi-quantitative total iron scoring system (range 0–60) described by Deugnier *et al*.^43^ in a blinded fashion. This scoring system accounts for iron deposition in three compartments: hepatocytic, sinusoidal and portal.

### Detection of cytokines in human sera

Cytokine concentrations in patient sera were quantified by multiplex protein arrays according to the manufacturer’s instructions (BioRad Laboratories, USA) as described recently^32^. In brief, 27-Plex and Bio-Plex Pro Human Cytokine 27-Plex (BioRad Laboratories) combined with HGF were used to detect IL-1β, IL-1RA, IL-2, IL-4, IL-5, IL-6, IL-7, IL-8/CXCL8, IL-9, IL-10, IL12(p70), IL-13, IL-15, IL-17, Eotaxin/CCL11, FGF-β, G-CSF, GM-CSF, IFN-γ, IP-10/CXCL10, MCP-1/CCL2, MIP-1a/CCL3, PDGF, MIP-1b/CCL4, RANTES/CCL5, TNF-α, VEGF, and HGF.

### Statistical analysis

The statistical analyses were performed with SPSS 15.0 and GraphPad Prism 5. The Mann-Whitney U test was used for comparisons of two groups and the Kruskal-Wallis test was used for comparisons of more than two groups. Fisher’s exact test was used for contingency tables. Correlation analyses were calculated with Spearman’s rank correlation. The Log rank test was used for the comparison of the cumulative treatment response rates. AUROC analyses and Youden’s index were used for the identification of cutoff values. P-values below 0.05 (two-tailed) were considered statistically significant in all analyses.

### Data availability

All data generated or analysed during this study are included in this published article (and its Supplementary Information files).

## Electronic supplementary material

Supplemental Information
