# Supplementary material for: Baseline IL-2 and the AIH score can predict the response to standard therapy in paediatric autoimmune hepatitis
Source: Sci Rep. 2018 Jan 11;8:419. doi: 10.1038/s41598-017-18818-5 (PMC5764983; doi:10.1038/s41598-017-18818-5)
Supplement: Supplementary file 1 — Supplemental Information [file 41598_2017_18818_MOESM1_ESM.pdf]

Baseline IL-2 and the AIH score can predict the response to standard therapy in paediatric autoimmune hepatitis.

[#]Jana Diestelhorst[1,2], [#]Norman Junge[2], Danny Jonigk[3], Jerome Schlue[3], Christine S. Falk[4], Michael P. Manns[1], Ulrich Baumann[2], *Elmar Jaeckel[1,5], *Richard Taubert[1,5]

[1] Department of Gastroenterology, Hepatology and Endocrinology, Hannover Medical School, Germany

[2] Pediatric Gastroenterology and Hepatology, Department of Paediatric Kidney, Liver and Metabolic Diseases, Hannover Medical School

[3] Institute for Pathology, Hannover Medical School, Hannover, Germany

[4] Institute of Transplant Immunology, Integrated Research and Treatment Center Transplantation (IFB-Tx), Hannover Medical School, Hannover, Germany

[5] Integrated Research and Treatment Center Transplantation (IFB-Tx), Hannover Medical School, Hannover, Germany

Supplementary Information

Supplementary Table 1: Additional data from paediatric AIH patients.

	Training cohort Median (IQR)	n	Validation cohort Median (IQR)	n
Age at diagnosis (years)	12.5 (6.4)	32	13.3 (5.4)	18
Gender (male/female)	6/26		6/12	
AIH type (type 1/type2)	27/5		18/0	
AIH score[a]	19.0 (5.0)	28	18.0 (4.0)	18
simplified AIH score[b]	7.0 (1.0)	29	7.0 (2.0)	18
**Autoantibodies**				
ANA[c]	24/32		16/17	
SMA[c]	25/32		15/17	
SLA	4/32		0/17	
LKM 1[c]	5/32		0/17	
pANCA	15/32		11/17	
**Laboratory test**				
IgG (times the ULN)	1.7 (1.4)	32	1.7 (0.8)	17
Alanine aminotransferase (times the ULN)	10.1 (16.1)	32	10.1 (22.6)	18
Aspartate aminotransferase (times the ULN)	12.2 (17.6)	32	15.8 (33.9)	18
Alkaline phosphatase (times the ULN)	1.0 (0.4)	32	1.1 (1.1)	18
Gamma-glutamyltransferase (times the ULN)	2.7 (3.1)	32	3.1 (8.4)	18
Bilirubin (times the ULN)	1.7 (4.0)	32	1.3 (4.6)	18
Prothrombin time (%)	73.0 (33.0)	31	72.0 (18.0)	18
**Iron homeostasis**				
Hb (g/dl)	12.5 (2.4)	28	12.7 (2.0)	16
Serum iron (µmol/l)	20.5 (27.3)	16	15.0 (18.0)	10
Transferrin saturation (%)	32.0 (32.0)	15	26.5 (24.0)	10
Iron binding capacity of transferrin (µmol/l)	69.0 (14.0)	17	68.5 (12.0)	10
Ferritin (times the ULN)	0.56 (1.64)	18	0.34 (1.56)	10
**Acute phase reactant**				
CRP (mg/l)	4.0 (4.6)	26	3.0 (5.0)	15
**Histology**				
mHAI	8.0 (7.5)	24	6.0 (3.0)	17
Fibrosis (Ishak)	4.0 (2.8)	24	3.0 (3.0)	17

[a] according to Alvarez et al.[1]; [b] according to Hennes et al.[2]; [c] according to AASLD guidelines 2010[3].

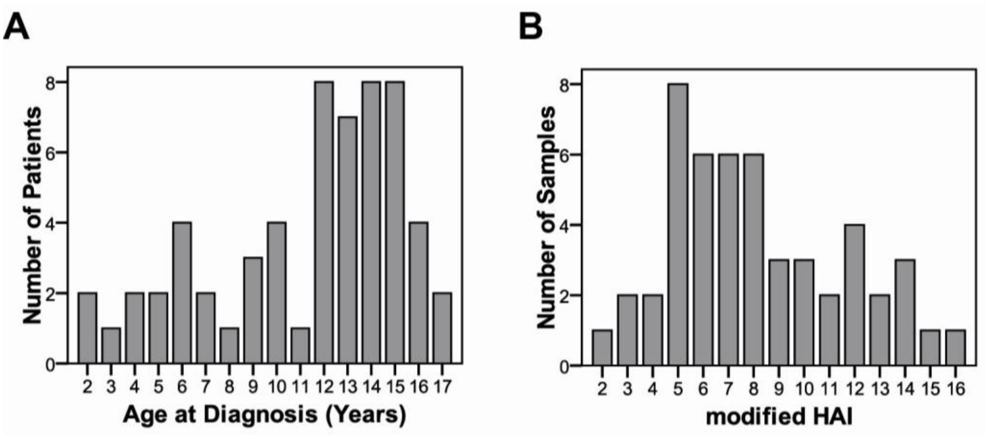

Supplementary Figure 1: Age and mHAI grades at the diagnosis of paediatric AIH.

(A) The age distribution of children at the diagnosis of AIH. (B) Scoring of mHAI of the liver biopsies prior to treatment.

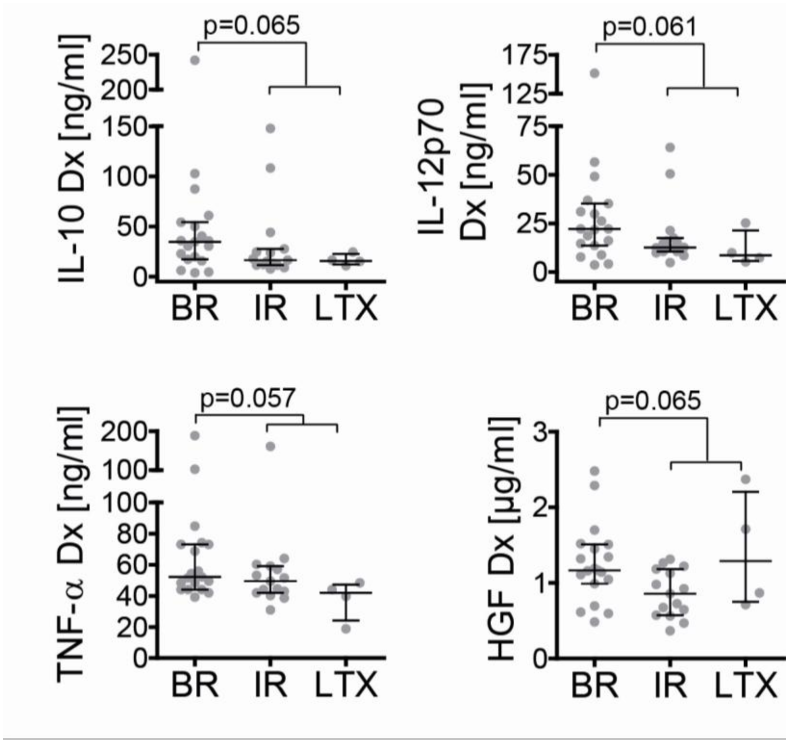

Supplementary Figure 2: Association of baseline cytokine levels with the subsequent treatment response in paediatric AIH.

Serum cytokine levels at diagnosis (Dx) in untreated pAIH patients with subsequent biochemical remission (BR: N=19), incomplete biochemical response (IR: N=15) and the need for liver transplantation (LTX: N=4). (HGF: hepatocyte growth factor)

References

1 Alvarez, F. *et al.* International Autoimmune Hepatitis Group Report: review of criteria for diagnosis of autoimmune hepatitis. *J Hepatol* **31**, 929-938 (1999).

2 Hennes, E. M. *et al.* Simplified criteria for the diagnosis of autoimmune hepatitis. *Hepatology* **48**, 169-176, doi:10.1002/hep.22322 (2008).

3 Manns, M. P. *et al.* Diagnosis and management of autoimmune hepatitis. *Hepatology* **51**, 2193-2213, doi:10.1002/hep.23584 (2010).
